# Genomic heterogeneity of historical gene flow between two species of newts inferred from transcriptome data

**DOI:** 10.1002/ece3.2152

**Published:** 2016-06-09

**Authors:** Michał T. Stuglik, Wiesław Babik

**Affiliations:** ^1^ Institute of Environmental SciencesJagiellonian UniversityGronostajowa 730387KrakowPoland

**Keywords:** Genomics, heterogeneous gene flow, introgression, *Lissotriton*, speciation

## Abstract

The role of gene flow in species formation is a major unresolved issue in speciation biology. Progress in this area requires information on the long‐term patterns of gene flow between diverging species. Here, we used thousands of single‐nucleotide polymorphisms derived from transcriptome resequencing and a method modeling the joint frequency spectrum of these polymorphisms to reconstruct patterns of historical gene flow between two *Lissotriton* newts: *L. vulgaris* (Lv) and *L. montandoni* (Lm). We tested several models of divergence including complete isolation and various scenarios of historical gene flow. The model of secondary contact received the highest support. According to this model, the species split from their common ancestor ca. 5.5 million years (MY) ago, evolved in isolation for ca. 2 MY, and have been exchanging genes for the last 3.5 MY Demographic changes have been inferred in both species, with the current effective population size of ca. 0.7 million in Lv and 0.2 million in Lm. The postdivergence gene flow resulted in two‐directional introgression which affected the genomes of both species, but was more pronounced from Lv to Lm. Interestingly, we found evidence for genomic heterogeneity of interspecific gene flow. This study demonstrates the complexity of long‐term gene flow between distinct but incompletely reproductively isolated taxa which divergence was initiated millions of years ago.

## Introduction

Speciation, which can be defined as the origin of reproductive barriers among populations (Coyne and Orr 2004), remains an essential problem in evolutionary biology ever since Darwin's time. Reproductive barriers can maintain genetic and phenotypic distinctiveness between populations in sympatry; hence, elucidating how they arise is crucial for the understanding of the formation of biodiversity (Seehausen et al. 2014). The presence and the role of gene flow during divergence are among major unresolved issues in speciation biology (Butlin et al. 2012; Abbott et al. 2013). If speciation proceeds without gene exchange between populations, reproductive isolation develops as a by‐product of selection and chance events, eventually resulting in completely isolated gene pools (Coyne and Orr 2004; Via 2009; Butlin et al. 2012; Sousa and Hey 2013). This process is relatively well understood, with multiple examples characterized to date (Dobzhansky 1937; Coyne and Orr 1997, 2004; Turelli et al. 2001).

However, in a history of divergence, ranges of incipient species often change, and thus, populations may experience periods of allopatry as well as recurrent contact resulting in gene exchange as long as reproductive isolation is not complete (Jiggins and Mallet 2000; Babik et al. 2003; Turner et al. 2005; Currat et al. 2008; Petit and Excoffier 2009; Martin et al. 2013; Nadachowska‐Brzyska et al. 2013; Zieliński et al. 2013, 2014). Reconstructing such historical processes – spanning hundreds of thousands or millions of years – constitutes a considerable challenge, complicated by the usual lack of a reliable record of range changes. It is not sufficiently understood how integrity of species is maintained in the face of gene flow, which is expected to homogenize diverging populations (Felsenstein 1981; Feder et al. 2012; Nosil and Feder 2012). These gaps in our understanding of the process of speciation can be filled using genomic data in suitable systems, along with new theoretical developments (Ellegren et al. 2012; Garrigan and Kingan 2012; Martin et al. 2013). For example, recently Feder et al. (2012) provided theory and predictions regarding genomewide patterns of divergence for speciation‐with‐gene‐flow. Their four‐stage model of the formation of reproductive isolation involves the processes of divergent selection, gene flow, and recombination. This model predicts that consecutive speciation phases are characterized by shifts in the genomic pattern of effective migration from nearly homogeneous toward heterogeneous, until complete genomic isolation, and thus, no genetic exchange occurs at the final stage. However the possible causes of genomic heterogeneity of divergence and its association with gene flow are currently hotly debated and the prospect for consensus in this area appears remote (Noor and Bennett 2009; Renaut et al. 2013; Cruickshank and Hahn 2014; Poelstra et al. 2014; Soria‐Carrasco et al. 2014; Burri et al. 2015).

**Figure 1:**
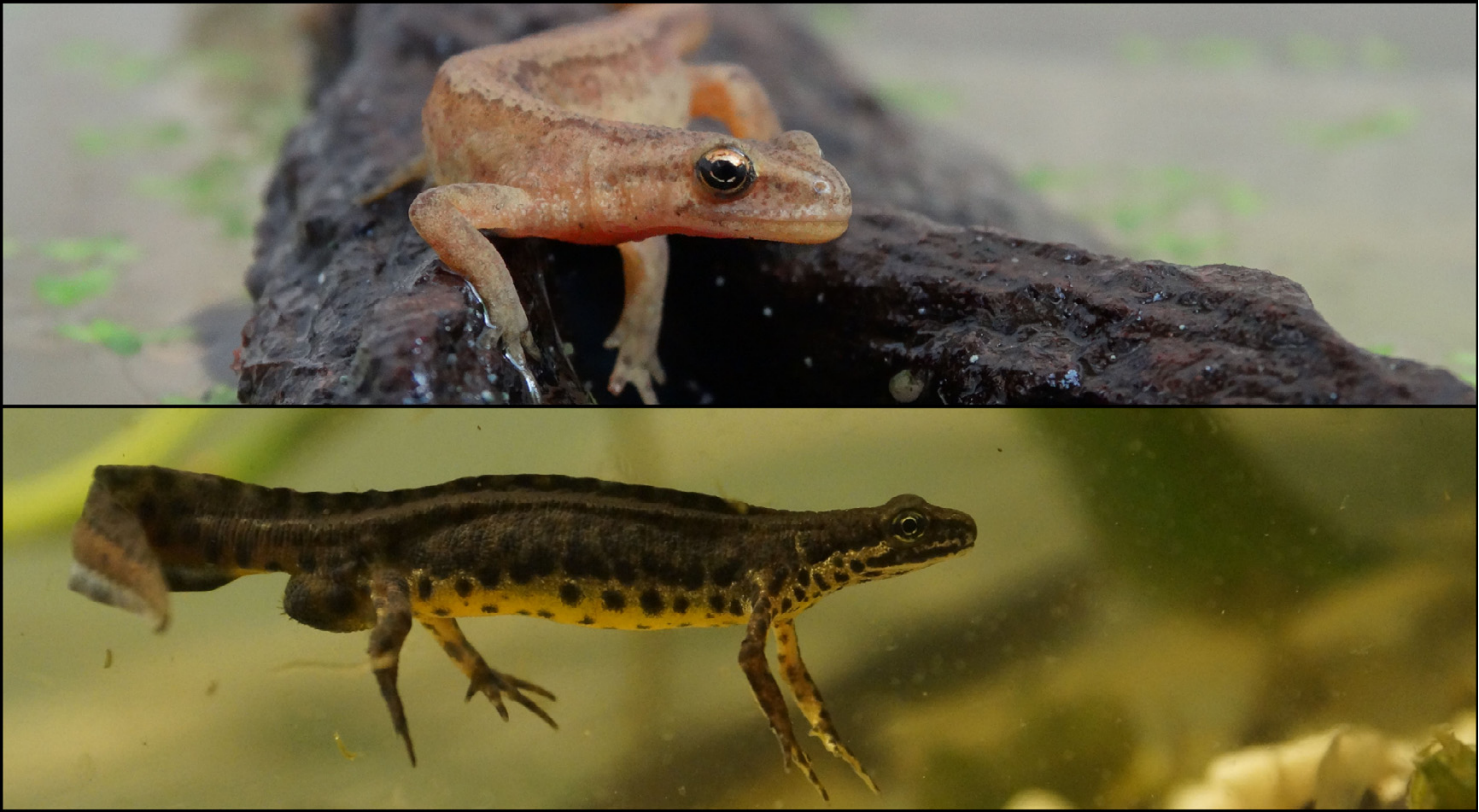
Examples of *Lissotriton montandoni* × *Lissotriton vulgaris* hybrids. Photograph: Marta Niedzicka.

Rapid development of new sequencing technologies (Metzker 2010; Mardis 2011; Shapiro et al. 2013) has provided access to various types of high‐throughput genetic markers. Together with newly developed computational methods, such data facilitate testing models of population divergence and inferring historical patterns of selection, introgression, divergence, and recombination across genome (Renaut et al. 2012; Sousa and Hey 2013). Here, we focus on two species of the genus *Lissotriton*, the Carpathian (*L. montandoni* Lm) and smooth (*L. vulgaris* Lv) newts (Fig. 1). The species diverged prior to the Pleistocene (Młynarski 1962, 1977; Hodrová 1987; Böhme and Ilg 2013). Currently, Lm and Lv have parapatric distributions and hybridize at lower mountain elevations in the Carpathians (Kotlík and Zavadil 1999; Babik et al. 2003; Zieliński et al. 2014). The distribution of genotypes and phenotypes in a hybrid zone is bimodal, implying substantial prezygotic isolation and possibly reinforcement of premating isolation in sympatry (Babik et al. 2003). Admixture in the nuclear genome only rarely extends beyond sympatry, as shown by the analyses based on allele frequencies in microsatellite and single‐nucleotide polymorphisms (SNP; Zieliński et al. 2013, 2014). However, the major histocompatibility complex (MHC) genes introgress more extensively, indicating genomic heterogeneity of interspecific gene flow (Nadachowska‐Brzyska et al. 2012). Perhaps the most dramatic manifestation of the impact of hybridization is seen in mtDNA, where complete replacement of the original Lm mtDNA occurred. Several mtDNA lineages currently found in Lm are entirely derived from Lv as a result of multiple, spatially and temporally distinct introgression events (Babik et al. 2005; Zieliński et al. 2013). This attests to a long history of genetic exchange between the two species, which may have profoundly affected their genomes.

Here, we use large amount of genomic data derived from transcriptome resequencing in the model‐based framework to test alternative scenarios and reconstruct the history of divergence and gene flow between Lm and Lv. We base the inferences on modeling the joint frequency spectrum of SNP utilizing the diffusion approximation. In particular, we wanted to (1) characterize intra‐ and interspecific genetic variation at the genomewide scale, (2) statistically test various hypotheses regarding the postdivergence patterns of gene flow including its genomic heterogeneity, (3) build a quantitative model of divergence, including temporal variation in gene flow and demography.

## Materials and Methods

### Sampling and sequencing

Liver tissue was obtained from 6 Lv (five localities) and 6 Lm (four localities) males (Figure S1). All sampled individuals were morphologically “pure,” with no evidence of hybrid origin. One individual of Lm (1998, Figure S1) was sampled in the pond where also Lv was found, the remaining individuals were samples from ponds in which no other *Lissotriton* species occurred. We sampled both Lv evolutionary lineages parapatric to Lm distribution (Zieliński et al. 2014). One *Lissotriton helveticus* (Lh) male was used as an outgroup. Total RNA was extracted from the liver tissue stored in RNA later using RNAzol RT (Molecular Research Center). RNA quality was assessed with Bioanalyzer; all samples had the RNA integrity number (RIN) > 7.0. RNAseq libraries were prepared using TruSeq RNA kit (Illumina, San Diego, California, United States). Libraries were sequenced on Illumina HiSeq2000, 2 × 100 bp reads (Table S1). Prior to de novo assembly raw reads were quality‐controlled with FastQC (Andrews 2010, version 0.10.1) and trimmed when necessary to remove low‐quality bases with the DynamicTrim, a script from the SolexaQA package (Cox et al. 2010).

### Transcriptome assembly and gene models

Low level of divergence between Lm and Lv allowed to use a common set of reference sequences, obtained by de novo assembly of liver transcriptome from 6 Lm individuals (167 million read pairs). Transcripts were reconstructed with Trinity transcriptome assembler (Grabherr et al. 2011), release 2012‐06‐08; with default parameters, except ‐‐bfly_opts “‐‐edge‐thr = 0.16”, which reduces the sensitivity of alternative splice detection. The Lh transcriptome was de novo assembled using 9.7 million of read pairs. De novo transcriptome assemblers commonly reconstruct as separate contigs divergent alleles within locus and alternatively spliced isoforms, although simulations show that many isoforms can be erroneously inferred (Vijay et al. 2013). In effect, de novo assembled transcriptome contains substantial redundancy: The same genomic sequence may be present in many contigs, which interferes with polymorphism detection. We removed this redundancy by constructing transcriptome‐based gene models (TGMs; Stuglik et al. 2014), separately for Lm (LmTGM) and Lh (LhTGM). TransDecoder pipeline included in the Trinity package was employed to identify putative coding regions (open reading frames, ORFs) in the TGM; minimum length of the coding sequence was set to 100 amino acids (Grabherr et al. 2011).

### Patterns of variation

We aligned all Lv and Lm reads to LmTGM with Bowtie 2 (Langmead and Salzberg 2012) with custom scoring function (‐‐score‐min L,−0.6, −0.44) increasing the minimum alignment score required for a valid alignment. SNP calling was performed with SAMtools pipeline, including mpileup, bcftools, and vcfutils (Li et al. 2009), with adjustment for highly covered genes (varFilter −D800000) and extended computation of base alignment quality (mpileup ‐Q 10 ‐ugDEf). Ancestral states were inferred by aligning the LhTGM sequences to LmTGM with GMAP (Wu and Watanabe 2005), and for orthologous mappings, we called genotypes for each position in SAMtools.

Patterns of variation were characterized at synonymous sites using the dataset constructed as follows (Var dataset). For each ORF, we created the unphased consensus sequence for each individual, considering as known only genotypes in positions: (1) covered by a minimum of 12 reads, (2) with genotype quality >= 20 Phred, and (3) with aligned outgroup (LhTGM) sequence; genotypes in the remaining positions were considered as missing data. Only loci with known genotypes covering minimum 70% of the ORF length (and a minimum of 35 full codons) in at least three individuals per species were included in the Var dataset. We excluded from all (i.e., Var and both Dem, see below) datasets genes: (1) derived from mitochondrial genome, (2) with high density of SNPs (>60 SNP/kb), and (3) the putative paralogos (i.e., loci with at least one position showing excessive heterozygosity (i.e., all six individuals heterozygous) in either species: probability of obtaining such genotype configuration for a biallelic locus is <0.05 assuming Hardy–Weinberg equilibrium and equal alleles frequencies).

To describe patterns of variation, we used the multiple alignments described above to calculate with MSCalc (Roux et al. 2011) a series of statistics at synonymous sites: nucleotide diversity (*π*), Watterson's *θ* (*θw*), Tajima's *D*,* F*_st_ (calculated as 1‐*π*_S_/*π*_T_, with *π*_S_ as the average nucleotide diversity within species and *π*_T_ as the total nucleotide diversity), mean interspecific divergence (*d*_XY_), and net interspecific divergence (*d*_A_). Additionally, we calculated for each gene the number of variable sites which were private for each species (Sx), shared between species (Ss), and fixed for different alleles in each species (Sf).

### Modeling demography

Demography was modeled using two datasets, each including only sites with genotypes callable for all individuals: (1) synonymous, containing only synonymous sites from putative protein‐coding loci (DemSyn dataset), (2) noncoding, containing sites outside ORFs in putative protein‐coding loci and all sites in putative noncoding loci (DemNcd dataset). To be included in the Site Frequency Spectrum (SFS), polymorphic sites must have fulfilled the following criteria for all individuals: (1) min. coverage 12×, (2) biallelic, (3) minimum overall SNP quality 30 Phred, (4) min. genotype quality 20 Phred, (5) established ancestral state (ancestral base had to segregate in the in‐group), (6) two bases adjoining SNP identical in all species (Lh, Lm, Lv) and nonpolymorphic. To minimize the effect of population structure within each species on demographic analyses, we used only a single individual from each locality (see Figure S1 for genetic structure and map of sampling sites) which resulted in 4 Lv and 5 Lm individuals, that is, 8 × 10 SFS.

We defined 16 demographic models and used ∂a∂i – diffusion approximations for demographic inference (Gutenkunst et al. 2009) – to estimate their likelihoods and parameter values. ∂a∂i models the joint SFS under various demographic processes, for example, expansions or migrations, it may also consider migration as a process heterogeneous across the genome. For a given model, the expected SFS was numerically calculated over a range of parameter values using the diffusion approximation of the population genetics of discrete number of individuals, evolving in discrete generations (Gutenkunst et al. 2009). Composite Poisson log‐likelihood and parameters values were estimated in the process of maximizing similarity between expected and observed, unfolded SFS. The BFGS algorithm was used as the method of likelihood optimization. Convergence was assured by multiple runs and random perturbations in initial parameter sets. We performed model selection by employing Bayesian information criteria to order models and adjusted likelihood ratio test (LRT) to compare models with the highest log‐likelihood values. In the LRT procedure, we used the Godambe information matrix to calculate adjustment to the *D* statistic. Then, we compared the adjusted *D* statistic to χ^2^ distribution with two degrees of freedom and calculated *P*‐value for this test (Coffman et al. 2016).

To investigate the joint demographic history of the two newt species, we compared 16 demographic models grouped into four classes of scenarios (Figure S2; Table S3). The models and scenarios were formulated taking into account the available information on the newt system and hypotheses formulated on the basis of previous research (Babik et al. 2005; Zieliński et al. 2013, 2014). All models assumed instantaneous split of the ancestral population into two descendant populations. Four classes of the considered scenarios were the following:

1. Strict isolation, that is, no gene flow after divergence; both constant (SI model) and variable sizes of descendant populations were considered (SIG model); in all variable size models, demographic changes were modeled as an exponential process.
2. Ancient migration (old gene flow); populations started to diverge in the presence of gene flow, but migration stopped after a period of time. We considered two genomic models of migration: (i) homogeneous, with a single migration rate in each direction shared by all loci (AM) and (ii) heterogeneous, with two rates of migration in each direction across the genome (AMHET); such heterogeneous model may represent two classes of loci: freely exchanged between species and those which exchange is opposed by selection. Homogeneous gene flow and heterogeneous gene flow were tested for each model allowing gene flow.
3. Secondary contact (recent gene flow), that is, gene flow followed a period of isolation after the initial divergence; (i) with constant demography (SC and SCHET models); (ii) with variable demography (SCG, SCGHET models); (iii) with variable demography only during the isolation period (SCIG, SCIGHET models); and (iv) with variable demography only during the migration period (SCMG, SCMGHET models).
4. Isolation with migration and continuous migration since the initial divergence, (i) with constant demography (IM, IMHET models) and (ii) variable demography (IMG, IMGHET models).

Each model was characterized by the parameter *T* (time of split, in units of 2*ancestral population size [*N*_A_] generations) and current population sizes for models with constant and variable demography. However, in models with ancient migration (AM, AMHET) and models of secondary contact (SC, SCHET, SCG, SCGHET, SCIG, SCIGHET, SCMG, SCMGHET), the time of split was a sum of two separately estimated parameters: durations of the isolation and migration periods. Gene flow was estimated separately in each direction, m12 denoted forward migration from population 2 into population 1 (i.e., m12 is the proportion of chromosomes per generation in population 1 that are new migrants from population 2), and m21 for migration in opposite direction. One set of such parameters was estimated for models with homogeneous gene flow (AM, SC, SCG, SCIG, SCMG, IM, IMG), but two for models with heterogeneous gene flow (AMHET, SCHET, SCGHET, SCIGHET, SCMGHET, IMHET, IMGHET). For secondary contact models with size change during isolation phase (SCG, SCGHET, SCIG, SCIGHET), we also estimated size of both populations after period of isolation. Moreover for SCG, SCGHET, SCIG, SCIGHET, IM, IMHET, IMG, IMGHET models, we estimated size (*s*) of population 1 immediately after the split as the fraction of the ancestral population size (size of 2nd populations was then 1 − s).

In order to convert parameters to biologically meaningful units, we estimated the ancestral population size (*N*_A_) using the formula *N*_A_ = *θ*/(4 *μ*L). The value of population mutation rate (*θ*) was estimated by ∂a∂i using the SFS (Theta_DemSyn_ = 8757; Theta_DemNcd_ = 14,967). Effective sequence length, that is, the number of resequenced bases with callable genotypes, here denoted as *L*, was estimated as 959,763 bp and 3,020,841 bp for DemSyn and DemNcd datasets, respectively. Mutation rate was calculated using sequence divergence from Lh at synonymous (0.058) and noncoding (0.036) sites, assuming the generation time of 4 years and the split between Lm/Lv and Lh 18.4 MY (Pabijan et al. 2015). The estimates of mutation rate were *u*_SYNONYMOUS_ = 6.3E‐09 and *u*_NONCODING_ = 3.9E‐09 per bp per generation.

Uncertainty of parameter estimation for SCGHET demographic model was assessed with conventional bootstrap (sampling with replacement over loci). We generated 120 resampled datasets and ran the inference procedure for each multiple times to assert stability during the process of optimization. Bootstrap confidence intervals were calculated as $\overset{¯}{\theta^{*}}\pm 1.96\sigma (\theta^{*})$, where ($\overset{¯}{\theta^{*}}$), *σ*(θ*) stand for mean and standard deviation of the parameter values estimated for the bootstrapped datasets, respectively. Parametric bootstrap was used to test goodness of fit of the best model (SCGHET). One‐hundred datasets were generated with ms (Hudson 2002) using the SCGHET model and its estimated parameters. The coalescent simulations were designed to follow as closely as possible the structure of the dataset used for calculation of the SFS; for each locus population, recombination rate was estimated using LDhat (McVean et al. 2002) and we assumed free recombination between loci. The comparison of the log‐likelihood and Pearson's χ^2^ of the data under the inferred model with the distributions of log‐likelihood and χ^2^ for simulated datasets provided the measure of the model fit.

## Results

### Patterns of nucleotide variation

**Figure 2:**
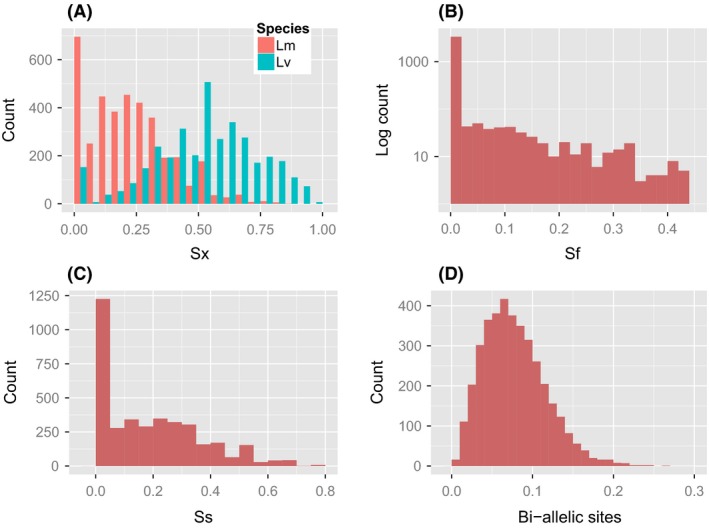
Patterns of variation at biallelic synonymous SNPs based on 3832 genes (Var dataset). Histograms show the following: (A) fraction of SNPs per gene that are exclusively polymorphic in either species (Sx); (B) fraction of SNPs per gene that represent fixed differences between species (Sf); (C) fraction of SNPs per gene shared by both species (Ss); (D) biallelic sites – fraction of SNPs per synonymous site.

**Figure 3:**
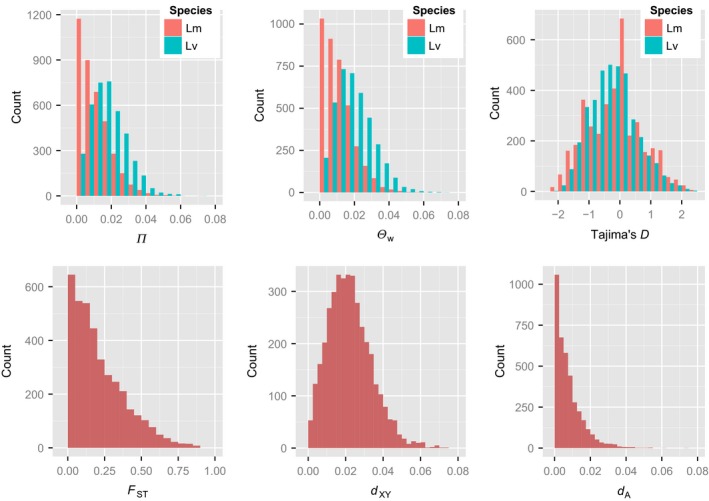
Patterns of synonymous polymorphism and divergence, based on 3832 genes (Var dataset). *π* – nucleotide diversity; *θ*_W_ – Watterson's theta; *d*_XY_ – mean pairwise sequence divergence; *d*_A_ – net sequence divergence.

Below, we present an overview of the patterns of variation and differentiation based on the Var dataset comprising 954 kb of synonymous sites located in 3832 putative protein coding TGMs (henceforth: genes) of six Lm and six Lv individuals (Figs. 2 and 3; Table 1). Of 73,786 synonymous biallelic SNPs, only 2% represented differences fixed between species, while shared polymorphisms constituted 20.7%, and those private to Lm and Lv 22.7% and 54.7%, respectively (Table 2). At least one fixed difference occurred in 12% (477) of genes, and 7% (281) genes had more than one fixed difference. The majority of genes (54.7%, 2097) contained more than one SNP shared between species. In accordance with the number of private polymorphisms, nucleotide diversity and Watterson's theta (averaged for all genes in dataset) were higher in Lv (*π*_Lv_ = 0.018 ±  [SD] 0.010, *θ*_WLv_ = 0.019 ± 0.011) than in Lm (*π*_Lm_ = 0.011 ±  [SD] 0.009, *θ*_WLm_ = 0.009 ± 0.011; Wilcoxon signed‐rank test, *V* = 6,237,333, *P* < 2.2E‐16 for *π*;* V* = 5,729,503, *P* < 2.2E‐16 for *θ*_W_). Higher overall polymorphism in Lv may reflect both larger effective population size and deeper genetic structuring of this species (Figs. 2 and 3; Table 1). The distribution of Tajima's *D* in both species was slightly shifted toward negative values, suggesting nonequilibrium demography and/or the influence of population structure and sampling scheme. The overall differentiation between species was moderate with the average *F*_ST_ of 0.217 ± 0.182, but variation among loci was high (Table 1; Fig. 3). Mean pairwise sequence divergence (*d*_*XY*_) was 0.022 ± 0.012, and the net sequence divergence (*d*_*A*_) was 0.008 ± 0.008 (Table 1; Fig. 3).

**Table 1:** Polymorphism and divergence at synonymous sites (Var dataset)

Statistic	Lv	Lm
π	0.018 ± 0.010	0.011 ± 0.009
*θ*_W_	0.019 ± 0.011	0.009 ± 0.011
Tajima's *D*	−0.251 ± 0.748	−0.146 ± 0.895
*F*_ST_	0.217 ± 0.182	
*d*_XY_	0.022 ± 0.012	
*d*_A_	0.008 ± 0.008	

Per gene mean ± standard deviation are given.

Lv, *Lissotriton vulgaris*; Lm, *L. montandoni*;* π*, nucleotide diversity; *θ*_W_, Watterson's theta; *d*_XY_, mean pairwise sequence divergence; *d*_A_, net sequence divergence.

**Table 2:** The number of synonymous single‐nucleotide polymorphisms (SNP) in various categories (Var dataset): Sf – fixed differences between species, Ss – polymorphisms shared by both species*,* Sx – polymorphisms private to one species; Lv – *Lissotriton vulgaris*, Lm – *L. montandoni*

SNP category	Number of SNP	Percentage of all SNPs
Sf (fixed)	1457	2.0
Ss (shared)	15,237	20.7
Lv Sx (private)	40,360	54.7
Lm Sx (private)	16,731	22.7
Total	73,786	100.0

### Historical demography and gene flow

We applied the diffusion approximation approach implemented in ∂a∂i to evaluate 16 models of historical demography and gene flow between Lm and Lv (Figure S2; Table S3). Data from four Lv and five Lm individuals were used, resulting in a 8 × 10 SFS. The models were evaluated using two datasets: DemSyn (47,236 synonymous SNPs located in 2942 genes) and DemNcd (91,904 noncoding SNPs in 3553 genes).

**Figure 4:**
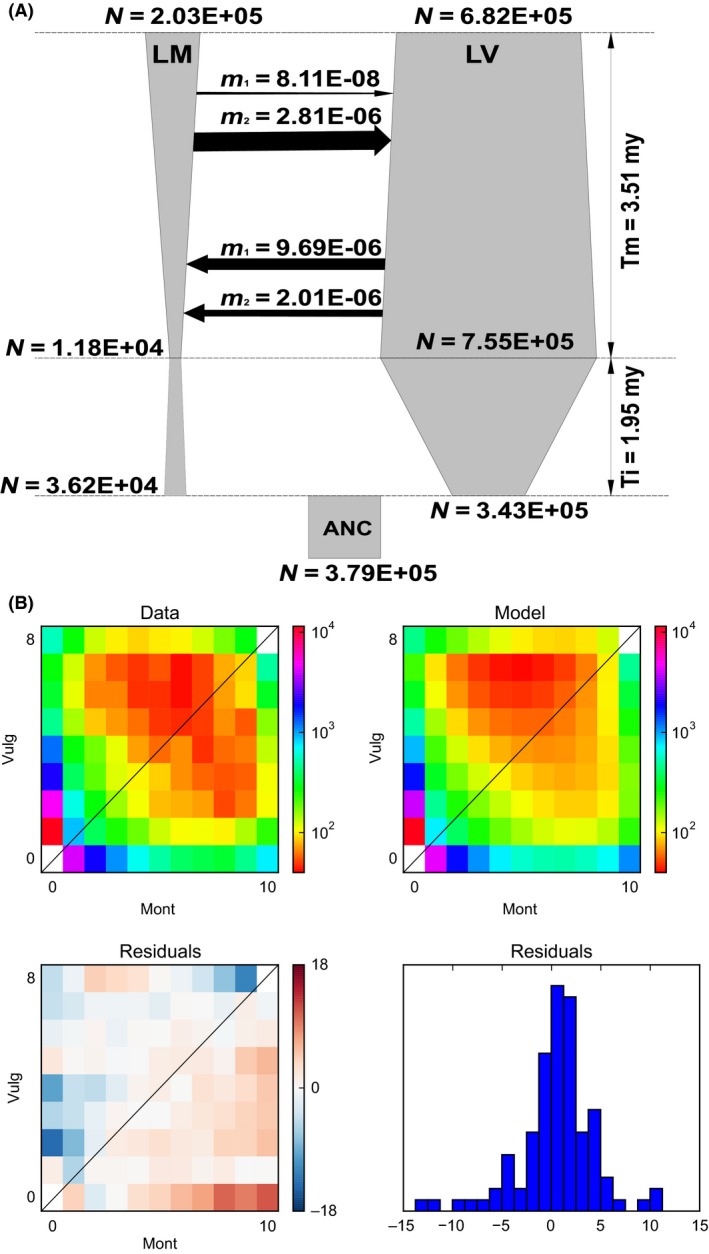
SCGHET model with estimated values of its parameters, where (A) shows migration both as a fraction of migrant chromosomes (number) and the effective number of immigrants (thickness of the arrow) per generation; m_1_ and m_2_ refer to the two rates of migration within the genome. *N* – effective population sizes. (B) Observed (data) and expected (model) site frequency spectra (SFS) under the SCGHET model based on synonymous SNPs. Cell i,j (i,j ‐ row, column index) represents the number of SNPs with i copies of derived allele in Lm and j copies of derived allele in Lv. Bottom row presents residual values between model and data. Red residuals indicate that the model predicts too many SNPs in a given cell, whereas blue indicates opposite.

Models assuming no postdivergence gene flow fitted the data poorly, while all models allowing for gene flow had better fit. Models with ancient migration consistently performed better, although still with low fit. Among models with gene flow, but assuming constant demography, model of secondary contact with heterogeneous gene flow (SCHET) was favored (Table S2). Overall, the best fit for both datasets was obtained by the model of secondary contact allowing demographic changes for both isolation and migration phases and heterogeneous gene flow across the genome (SCGHET; Table S2; Fig. 4). The best fit of the SCGHET model was confirmed by the adjusted log‐LRT comparing it with the SCIGHET model (*P*_DemSym_ < 4E‐5; *P*_DemNcd_ < 3.5E‐7). The accuracy of demographic inferences was evaluated for the best SCGHET model. The parametric bootstrap analysis and goodness‐of‐fit test showed that even this best of the evaluated models did not adequately explain all features of the data (Figures S3–S6). This indicates that the actual process of divergence and gene flow was more complex than assumed by the relatively simple models evaluated here.

### Divergence history

Parameters of the SCGHET model estimated from both the DemSyn and DemNcd datasets were largely concordant (Table 3), so below we present only the results obtained for the synonymous dataset. An ancestral population of the effective size of ca. 0.38 million individuals split ca. 5.5 MY unevenly into Lm and Lv; only a minor fraction of the ancestral population, estimated as 9.5%, founded Lm. After the initial split, during a ca. 2 MY period of isolation, Lm experienced population decline, whereas demographic growth occurred in Lv (Table 3). At ca. 3.5 MY, bidirectional and heterogeneous across the genome gene flow started. In about 2/5 of the genome, migration rate was of comparable magnitude (ca. 2E‐06 per gene copy) in both directions, while in the remaining 3/5 of the genome, migration rate was lower and asymmetric: migration into Lm was an order of magnitude higher (ca. 1E‐07) than into Lv (ca. 1E‐08). When gene flow was measured as the effective number of migrants, which may be easier to interpret in biological terms, the picture was somewhat different due to the differences in the effective population sizes of the recipient species (Table 3). After the onset of gene flow, effective population size of Lv has been relatively stable, while Lm experienced considerable demographic expansion (Table 3).

**Table 3:** Maximum likelihood (ML) parameter estimates for the SCGHET model. The 95% confidence intervals (CI) were obtained by conventional bootstrap over loci. Lv – *Lissotriton vulgaris*, Lm – *L. montandoni*, S – fraction of the ancestral population that founded Lm, N_A_ – ancestral effective population size, Ne – effective population size; *T*_i_ – duration of the isolation period (years); *T*_m_ – duration of the gene exchange period (years); m2Lv – fraction of individuals each generation in Lv that are new migrants from Lm; m2Lm – fraction of individuals each generation in Lm that are new migrants from Lv; (1), (2) – categories of migration rate; P – fraction of migrating genes for category (1), fraction of migrating genes for category (2) is 1−P

Parameter	ML estimate	95% LCI	95% HCI
DemSyn dataset (synonymous polymorphisms)			
*S*	0.10	0.02	0.16
*N*_A_	3.79E+05	3.66E+05	3.91E+05
Lv Ne at the start of migration period	7.55E+05	5.26E+05	9.44E+05
Lm Ne at the start of migration period	1.18E+04	3.71E+03	2.18E+04
Lv Ne at present	6.82E+05	5.28E+05	9.07E+05
Lm Ne at present	2.03E+05	1.59E+05	2.85E+05
*T*_i_ duration of the isolation period (years)	1.95E+06	8.03E+05	2.77E+06
*T*_m_ duration of the migration period (years)	3.51E+06	2.85E+06	5.61E+06
Time of divergence (*T*_i_ + *T*_m_) (years)	5.46E+06	–	–
m2Lv (1)	8.11E‐08	3.98E‐08	1.20E‐07
m2Lm (1)	9.69E‐07	6.05E‐07	1.20E‐06
Effective migration rate into Lv (1)	0.06	0.03	0.09
Effective migration rate into Lm (1)	0.20	0.15	0.25
m2Lv (2)	2.81E‐06	2.03E‐06	3.18E‐06
m2Lm (2)	2.01E‐06	1.20E‐06	2.56E‐06
Effective migration rate into Lv (2)	1.92	1.29	2.44
Effective migration rate into Lm (2)	0.41	0.24	0.59
*P*	0.62	0.47	0.75
DemNcd dataset (noncoding polymorphisms)			
*S*	0.09	0.02	0.13
*N*_A_	3.17E+05	3.04E+05	3.30E+05
Lv Ne at the start of migration period	6.82E+05	4.76E+05	7.86E+05
Lm Ne at the start of migration period	1.97E+04	4.30E+03	3.84E+04
Lv Ne at present	5.87E+05	4.77E+05	8.18E+05
Lm Ne at present	2.29E+05	1.71E+05	2.87E+05
*T*_i_ duration of the isolation period (years)	1.11E+06	4.51E+05	1.72E+06
*T*_m_ duration of the migration period (years)	3.57E+06	2.57E+06	4.68E+06
Time of divergence (*T*_i_ + *T*_m_) (years)	4.68E+06	–	–
m2Lv (1)	4.86E‐08	2.32E‐08	7.58E‐08
m2Lm (1)	7.15E‐07	4.80E‐07	1.00E‐06
Effective migration rate into Lv (1)	0.03	0.01	0.05
Effective migration rate into Lm (1)	0.16	0.12	0.22
m2Lv (2)	2.74E‐06	1.55E‐06	3.81E‐06
m2Lm (2)	1.53E‐06	6.10E‐07	2.57E‐06
Effective migration rate into Lv (2)	1.61	0.96	2.48
Effective migration rate into Lm (2)	0.35	0.14	0.58
*P*	0.66	0.59	0.84

## Discussion

The two most important findings of this study are long‐term genetic exchange following a substantial period of isolation between Lv and Lm and genomic heterogeneity of gene flow between them. Thus, by providing information about the amount, timing, and variation in gene flow, the study contributes to the ongoing debate about the impact of gene flow on the process of speciation (Seehausen 2004; Abbott et al. 2013; Barton 2013; Strasburg and Rieseberg 2013; Larson et al. 2014).

The best‐fitting model indicates that divergence was initiated without gene flow, possibly in allopatry. However, genomewide gene flow has been a prominent feature of the Lm/Lv system over an extended period of time. Even the best‐fitting model is relatively simple and does not adequately explain all features of the data, and hence, it undoubtedly oversimplifies the complex history of genetic exchange between the two species. For instance, even prolonged isolation within the Pleistocene may remain undetected, because only single periods of isolation and gene flow were allowed. Nevertheless the parameters of the best‐fitting secondary contact model indicate that the newts experienced rather long‐term genetic exchange than just episodic bouts of gene flow. Our results are consistent with the view that completion of reproductive isolation may take hundreds of thousands of generations (Butlin 2005; Pinho and Hey 2010; Abbott et al. 2013).

The genomewide nuclear perspective provided by the ∂a∂i models is supported by the mtDNA data. Multiple, spatially and temporally separated introgression events from Lv which led to the complete replacement of the original Lm mtDNA attest to the long‐term genetic exchange between species (Babik et al. 2005; Zieliński et al. 2013). While the origin of the mtDNA lineage I was dated to ca. 0.7 MY (Pabijan et al. 2015), which may coincide with the time of introgression, introgression of two other mtDNA lineages currently common in both species, G and J, was dated to the last glacial period (Zieliński et al. 2013). Given the long‐term genetic exchange and relatively low migration rate detected in the present study, it is likely that although reproductive isolation is not complete, speciation is irreversible and species are able to maintain their integrity in sympatry.

Nuclear sequence data were used to investigate historical gene flow between Lv and Lm in another recent study (Zieliński et al. (in press)), which drew a similar overall picture, but some differences regarding history of gene flow deserve discussion. Zieliński et al. also found support for secondary contact but their models limit secondary contact to the last 200 kya or the last two glacial periods. The secondary contact model of Zieliński et al. scored better than the secondary contact model allowing also an older admixture, which is probably more comparable to the secondary contact model evaluated in the present study. Thus, the work of Zieliński et al. suggests more intensive genetic exchange more recently. The results of these two modeling studies are difficult to compare directly for several reasons: (1) different analytical methods, SFS‐based vs. Approximate Bayesian Computations (ABC) and related differences in the details of model specification, (2) sample sizes and geographic extent of sampling, (3) including vs. not including genomic heterogeneity of introgression in the models. However, these differences increase our confidence in the findings common to both studies. Thus, there is robust evidence for periods of isolation and genetic exchange between Lv and Lm during the history of divergence. Considerable uncertainty remains, however, with respect to whether genetic exchange has been intensive for prolonged periods or whether it has been more episodic.

The results from historical models and mtDNA may appear at odds with restricted contemporary gene flow found in a hybrid zone (Babik et al. 2003), in analyses of admixture at a broader geographic scale (Zieliński et al. 2013, 2014) and in laboratory tests of behavioral reproductive isolation (Michalak et al. 1997; Michalak and Rafinski 1999). Although hybridization and introgression do occur in the hybrid zone and F1 hybrids are highly fertile in the laboratory (M. Niedzicka and W. Babik, unpubl. data), reproductive isolation is strong and nuclear introgression does not extend beyond sympatry, with exception of some marginal Lm populations (Zieliński et al. 2014). How to reconcile these seemingly contradictory findings? First, it is possible that indeed current gene flow does not extend beyond sympatry because of a genomewide barrier formed by the stable hybrid zones (Barton and Bengtssont 1986) and gene flow inferred from statistical models actually occurred in a recent past. For example, massive neutral introgression is predicted following range expansions and invasions which probably accompanied major climatic perturbations of the late Pleistocene (Currat et al. 2008; Excoffier et al. 2009). On the other hand, it is also possible that the levels of gene flow difficult to detect in studies looking at admixture in contemporary populations are sufficient to maintain signal of long‐term gene flow detectable in genetic data. For example, Sambatti et al. (2012) found that although reproductively successful hybrids between two sunflower species are produced at the rate of 10^−4^–10^−6^, the number of hybrids and the effect of hybridization on genomes of hybridizing species may be large, because of their large effective population sizes. The long‐term effective population sizes of the studied newts are on the order of hundreds of thousands. Therefore, even the relatively low migration rates detected in the current study result in appreciable number of migrants exchanged between species, which, as theory predicts, determines the impact of gene flow on genetic composition of hybridizing species.

The second major finding of the current study was substantial genomewide heterogeneity of gene flow. The model assuming two classes of loci differing in the strength of gene flow had considerably better fit than the model assuming a single migration rate. At approximately 40% of the genome, the rates of interspecific gene flow appear similar in both directions, while in the remaining 60%, gene flow has been strongly constrained and asymmetric, an order of magnitude stronger from Lv to Lm than vice versa. Genomic heterogeneity was already suggested by Zieliński (Zieliński et al. 2014), but here, we provide a truly genomewide evidence. These findings add to the growing number of studies (Martin et al. 2013; Roux et al. 2013, 2014; Tine et al. 2014; Fontaine et al. 2015; Lamichhaney et al. 2015) showing the persistence and genomewide heterogeneity of interspecific gene flow even long after the divergence was initiated. Such pattern is consistent with advanced, but not final, stages of speciation (Wu 2001; Via 2009; Feder et al. 2012).

Heterogeneity of introgression is inferred from heterogeneity of divergence. For example, in the method employed here, under the model of genomic heterogeneity, SFS is assumed to be composed of two spectra differing in the amount of shared polymorphism. There is, however, an ongoing debate about the mechanisms causing heterogeneous genomic divergence, and one should therefore carefully consider alternative mechanisms capable of explaining the observed pattern. One such explanation, implicit in the concept of genomic heterogeneity of gene flow, is that numerous loci involved in the Bateson–Dobzhansky–Muller incompatibilities and/or under divergent selection accumulated across the genome, but their distribution is heterogeneous enough to produce differences in the propensity to introgression (Bierne et al. 2011; Soria‐Carrasco et al. 2014). Another viable explanation advocated by several recent studies is that the differences in the amount of polymorphism shared across the genome may result from features of genome architecture, for example, its recombination landscape (Ellegren et al. 2012; Renaut et al. 2013; Cruickshank and Hahn 2014). Unfortunately, the importance of these two mechanisms in the newt system cannot be easily tested with the available data. However, it should be feasible to disentangle these two mechanisms in a study of replicated transects through Lm × Lv hybrid zones.

A special example of genomic heterogeneity of introgression between Lm and Lv is provided by the MHC class II genes, which introgress more extensively than microsatellite loci (Nadachowska‐Brzyska et al. 2012). However, MHC genes may be outliers in this respect because they evolve under balancing selection, which commonly operates through the mechanism of rare allele advantage. Under such mechanism, initially rare introgressed alleles may be easily established in the recipient species because they are favored by selection (Schierup et al. 2000; Castric et al. 2008). The case of MHC genes illustrates a more general phenomenon: as long as some interspecific gene flow occurs, variants emerging in one species and conferring adaptive advantage to both can easily spread leading to the effective sharing of adaptations between species (Piálek and Barton 1997; Barton 2001; Rieseberg et al. 2003; Fitzpatrick et al. 2010).

A potential limitation of the present study which deserves attention is the sampling scheme in relation to the assumptions of the models used. Amphibians and particularly urodeles often show substantial and old intraspecific genetic structure (Vences and Wake 2007). In our case, two deeply diverged evolutionary lineages of Lv come into contact with Lm within and outside the Carpathian Basin (Babik et al. 2005; Zieliński et al. 2013, 2014). Unfortunately, we were not able to apply three‐population models due to the small number of individuals sampled. Thus, our study reconstructs the history of gene flow effectively averaged over the two groups of Lv. Gene flow between Lm and Lv in the context of genetic structuring of the latter was explored by Zieliński et al. (in press). Because methods used for demographic inferences often assume panmixia within species, even finer scale genetic structuring may affect their performance and bias results. Newts form discrete demes corresponding to breeding ponds and their regional populations can be considered meta‐populations (Marsh and Trenham 2001; Smith and Green 2005). It has been shown (Wakeley 1999, 2004; Wakeley and Aliacar 2001) that if one gene copy per locus is sampled per deme, ancestral process producing such sample is identical to the unstructured coalescent process, if time is rescaled appropriately. The site frequency spectrum obtained from such samples would then effectively remove the effect of population structure. With limitation of our sampling in terms of the number of individuals, we could not fully apply this recommendation, but to obtain a more balanced sample for demographic inferences, we constructed the SFS using one individual per population. The expected effect of such procedure on the SFS would be an excess of doubletons, which was indeed observed. Both pooling of two evolutionary lineages within the Lv and sampling of two, instead of one gene copy per population may contribute to the imperfect fit of the data to the demographic model.

## Data Accessibility

Raw reads were deposited in BioProject portal (PRJNA316531, PRJNA316537, PRJNA316561). Transcriptome contigs, variant calling (VCF) files were deposited in Dryad Digital Repository entry doi:10.5061/dryad.bm4nd

## Conflict of Interest

None declared.

## Supporting information


**Table S1.** Number of raw reads; number and percentage of reads aligned to LmTGM.
**Table S2.** Comparison of 16 demographic models.
**Table S3.** Comparison of 16 demographic models. Common features highlighted with “+” sign.
**Figure S1.** Distribution of sampling localities.
**Figure S2.** Demographic models.
**Figure S3.** Goodness‐of‐fit tests for DemSyn dataset based on 100 parametric bootstraps with better fits placed closer to zero.
**Figure S4.** Goodness‐of‐fit tests for DemNcd dataset based on 100 parametric bootstraps with better fits placed closer to zero.
**Figure S5.** SCGHET model conventional bootstrap results. Distribution of model's parameters estimates based on 120 simulated DemSyn datasets, generated using maximum likelihood values from the real data (blue lines).
**Figure S6.** SCGHET model conventional bootstrap results. Distribution of model's parameters estimates based on 120 simulated DemNcd datasets, generated using maximum likelihood values from the real data (blue lines).
