# Supplementary material for: Genomic heterogeneity of historical gene flow between two species of newts inferred from transcriptome data
Source: Ecol Evol. 2016 Jun 9;6(13):4513–25. doi: 10.1002/ece3.2152 (PMC4930998; doi:10.1002/ece3.2152)
Supplement: Supplementary file 1 — Table S1. Number of raw reads; number and percentage of reads aligned to LmTGM. Table S2. Comparison of 16 demographic models. Table S3. Comparison of 16 demographic models. Common features highlighted with “+” sign. Figure S1. Distribution of sampling localities. Figure S2. Demographic models. Figure S3. Goodness‐of‐fit tests for DemSyn dataset based on 100 parametric bootstraps with better fits placed closer to zero. Figure S4. Goodness‐of‐fit tests for DemNcd dataset based on 100 parametric bootstraps with better fits placed closer to zero. Figure S5. SCGHET model conventional bootstrap results. Distribution of model's parameters estimates based on 120 simulated DemSyn datasets, generated using maximum likelihood values from the real data (blue lines). Figure S6. SCGHET model conventional bootstrap results. Distribution of model's parameters estimates based on 120 simulated DemNcd datasets, generated using maximum likelihood values from the real data (blue lines). [file ECE3-6-4513-s001.docx]

Genomic heterogeneity of historical gene flow between two species of newts inferred from transcriptome data - Supporting Information

Michał T. Stuglik* & Wiesław Babik

# Supporting Tables

### Supporting Table 1. Number of raw reads; number and percentage of reads aligned to LmTGM.

| **Species** | **Sample ID** | **Number of reads** | **Number of reads aligned to LmTGM** | **Percentage of reads aligned to LmTGM** |
| --- | --- | --- | --- | --- |
| Lv | 1884 | 72 592 038 | 54 599 159 | 75,2 |
|  | 1925 | 64 199 182 | 47 817 220 | 74,5 |
|  | 1926 | 58 608 206 | 44 219 648 | 75,4 |
|  | 1961 | 57 615 108 | 43 093 675 | 74,8 |
|  | 2389 | 67 059 688 | 49 768 793 | 74,2 |
|  | 2527 | 70 285 062 | 53 301 099 | 75,8 |
|  | Total | 390 359 284 | 292 799 594 | 75,0 |
|  | Mean | 65 059 881 | 48 799 932 |  |
|  | SD | 4 919 049 | 3 756 418 |  |
|  | Median | 65 629 435 | 48 793 007 |  |
| Lm | 1940 | 57 884 142 | 44 369 949 | 76,7 |
|  | 1941 | 52 147 866 | 40 860 643 | 78,4 |
|  | 1998 | 44 794 482 | 34 822 202 | 77,7 |
|  | 2386 | 54 153 152 | 42 080 877 | 77,7 |
|  | 2517 | 61 346 258 | 48 437 422 | 79,0 |
|  | 2530 | 65 096 410 | 50 390 429 | 77,4 |
|  | Total | 335 422 310 | 260 961 522 | 77,8 |
|  | Mean | 55 903 718 | 43 493 587 |  |
|  | SD | 5 538 552 | 4 239 013 |  |
|  | Median | 56 018 647 | 43 225 413 |  |
| Lh |  | 15 522 976 | - | - |

### Supporting Table 2. Comparison of 16 demographic models. BIC - Bayesian information criterion (BIC), ΔBIC - difference between the BIC value for the given model and the best model.

| **DemSyn dataset (synonymous polymorphisms)** | | | | |
| --- | --- | --- | --- | --- |
| **Model** | **No. parameters** | **Log-likelihood** | **BIC** | **Δ BIC** |
| SI | 3 | -3602 | 7228 | 4970 |
| SIG | 4 | -3551 | 7133 | 4875 |
| AMHET | 9 | -2487 | 5046 | 2788 |
| AM | 6 | -1790 | 3627 | 1369 |
| IMHET | 8 | -1748 | 3561 | 1303 |
| IM | 5 | -1729 | 3498 | 1240 |
| SC | 6 | -1664 | 3376 | 1118 |
| IMG | 6 | -1556 | 3160 | 902 |
| SCMG | 8 | -1490 | 3044 | 786 |
| SCHET | 9 | -1440 | 2952 | 694 |
| SCIG | 7 | -1389 | 2835 | 577 |
| SCG | 9 | -1363 | 2798 | 540 |
| SCMGHET | 11 | -1332 | 2752 | 494 |
| IMGHET | 9 | -1216 | 2504 | 246 |
| SCIGHET | 10 | -1189 | 2458 | 200 |
| SCGHET | 12 | -1081 | 2258 |  |
| **DemNcd dataset (noncoding polymorphisms)** | | | | |
| **Model** | **No. parameters** | **Log-likelihood** | **BIC** | **Δ BIC** |
| SI | 3 | -8524 | 17073 | 12106 |
| SIG | 4 | -8411 | 16853 | 11886 |
| AMHET | 9 | -6302 | 12676 | 7709 |
| IMHET | 8 | -4083 | 8230 | 3263 |
| AM | 6 | -4074 | 8195 | 3228 |
| IM | 5 | -3959 | 7958 | 2991 |
| SC | 6 | -3805 | 7658 | 2691 |
| SCHET | 9 | -3438 | 6948 | 1981 |
| IMG | 6 | -3297 | 6642 | 1675 |
| SCMG | 8 | -3254 | 6572 | 1606 |
| SCIG | 7 | -3124 | 6304 | 1337 |
| SCMGHET | 11 | -2914 | 5916 | 949 |
| IMGHET | 9 | -2729 | 5529 | 562 |
| SCG | 9 | -2699 | 5470 | 503 |
| SCIGHET | 10 | -2662 | 5404 | 437 |
| SCGHET | 12 | -2436 | 4967 |  |

Models were grouped into for classes according to the gene flow scenarios: (1) SI - strict isolation; SIG - strict isolation with demographic changes; (2) AM - ancient migration; AMHET - ancient migration, heterogeneous gene flow; (3) SC - secondary contact; SCHET - secondary contact, heterogeneous gene flow; SCG - secondary contact, demographic changes; SCGHET - secondary contact, heterogeneous gene flow, demographic changes; SCMG - secondary contact, demographic changes in migration phase; SCMGHET - secondary contact, heterogeneous gene flow, demographic changes in migration phase; SCIG - secondary contact, demographic changes in isolation phase; SCIGHET - secondary contact, heterogeneous gene flow, demographic changes in isolation phase; (4) IM - isolation with migration; IMG - isolation with migration, continuous migration, demographic changes; IMHET - isolation with migration, heterogeneous gene flow; IMGHET - isolation with migration, heterogeneous gene flow, demographic changes.

### Supporting Table 3. Comparison of 16 demographic models. Common features highlighted with “+” sign.

| **Model** | **No. parameters** | **Strict isolation** | **Ancient migration** | **Secondary contact** | **Continuous migration** | **Heterogeneous gene flow** | **Homogeneous gene flow** | **Demographic changes** |
| --- | --- | --- | --- | --- | --- | --- | --- | --- |
| SI | 3 | + |  |  |  |  |  |  |
| SIG | 4 | + |  |  |  |  | + | + |
| AMHET | 9 |  | + |  |  | + |  |  |
| AM | 6 |  | + |  |  |  | + |  |
| SC | 6 |  |  | + |  |  | + |  |
| SCHET | 9 |  |  | + |  | + |  |  |
| SCG | 9 |  |  | + |  |  | + | + |
| SCGHET | 12 |  |  | + |  | + |  | + |
| SCIG | 7 |  |  | + |  |  | + | + |
| SCIGHET | 10 |  |  | + |  | + |  | + |
| SCMG | 8 |  |  | + |  |  | + | + |
| SCMGHET | 11 |  |  | + |  | + |  | + |
| IM | 5 |  |  |  | + |  | + |  |
| IMHET | 8 |  |  |  | + | + |  |  |
| IMG | 6 |  |  |  | + |  | + | + |
| IMGHET | 9 |  |  |  | + | + |  | + |

Models were grouped into for classes according to the gene flow scenarios: (1) SI - strict isolation; SIG - strict isolation with demographic changes;

(2) AM - ancient migration; AMHET - ancient migration, heterogeneous gene flow; (3) SC - secondary contact; SCHET - secondary contact, heterogeneous gene flow; SCG - secondary contact, demographic changes; SCGHET - secondary contact, heterogeneous gene flow, demographic changes; SCMG - secondary contact, demographic changes in migration phase; SCMGHET - secondary contact, heterogeneous gene flow, demographic changes in migration phase; SCIG - secondary contact, demographic changes in isolation phase; SCIGHET - secondary contact, heterogeneous gene flow, demographic changes in isolation phase; (4) IM - isolation with migration; IMG - isolation with migration, continuous migration, demographic changes; IMHET - isolation with migration, heterogeneous gene flow; IMGHET - isolation with migration, heterogeneous gene flow, demographic changes.

# Supporting Figures

### Supporting Figure 1. Distribution of sampling localities. Purple dots – *Lissotriton vulgaris*; black squares – *Lissotriton montandoni*; hatched area - Lm species range. Inset shows the neighbor joining tree based on the average *p* distances between individuals; tree scale: 10000 differences equal 0.00175 divergence per site; each internode received 100% bootstrap support.

**
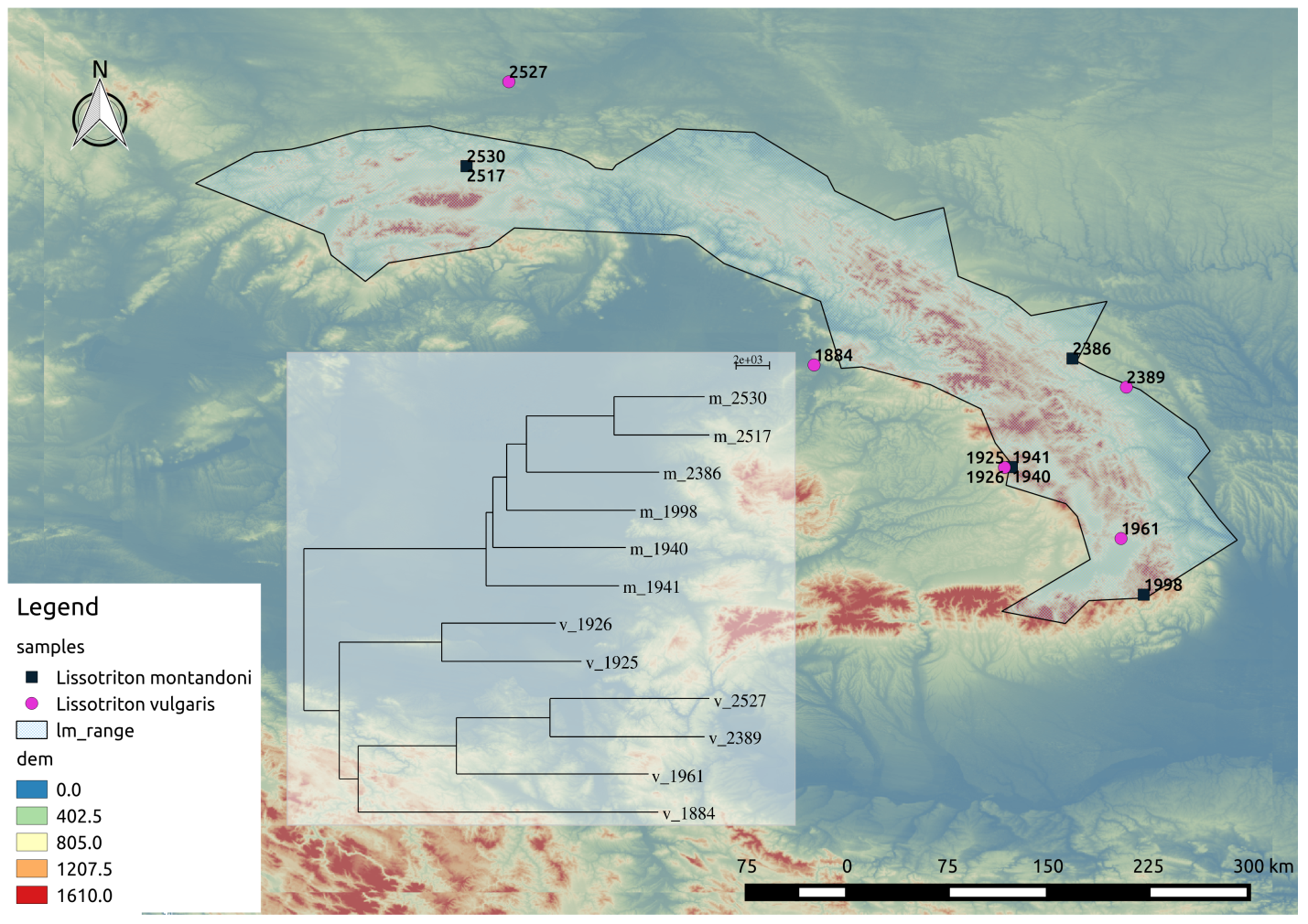
**

### Supporting Figure 2. Demographic models.


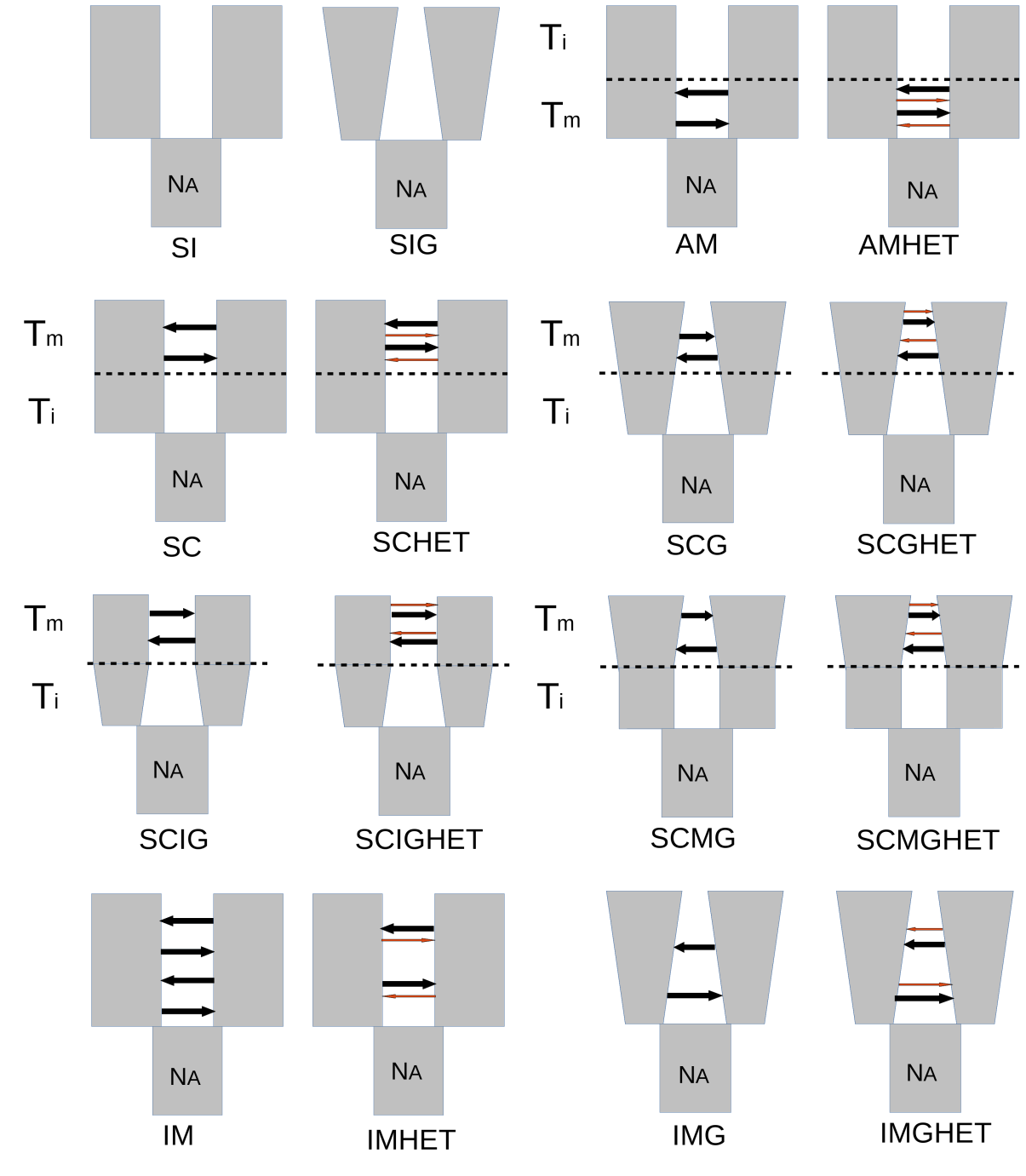


Models’ name within four classes of scenario: (1) SI - strict isolation; SIG - strict isolation with demographic changes;

(2) AM - ancient migration; AMHET - ancient migration, heterogeneous gene flow; (3) SC - secondary contact; SCHET - secondary contact, heterogeneous gene flow; SCG - secondary contact, demographic changes; SCGHET - secondary contact, heterogeneous gene flow, demographic changes; SCMG - secondary contact, demographic changes in migration phase; SCMGHET - secondary contact, heterogeneous gene flow, demographic changes in migration phase; SCIG - secondary contact, demographic changes in isolation phase; SCIGHET - secondary contact, heterogeneous gene flow, demographic changes in isolation phase; (4) IM - isolation with migration; IMG - isolation with migration, continuous migration, demographic changes; IMHET - isolation with migration, heterogeneous gene flow; IMGHET - isolation with migration, heterogeneous gene flow, demographic changes.

### Supporting Figure 3. Goodness-of-fit tests for DemSyn dataset based on 100 parametric bootstraps with better fits placed closer to zero. (A) Distribution of log-likelihoods for simulations (red) with value from real data set (blue). (B) Distribution of Pearson’s X2 statistic for simulations (red) with value from real data set (blue).


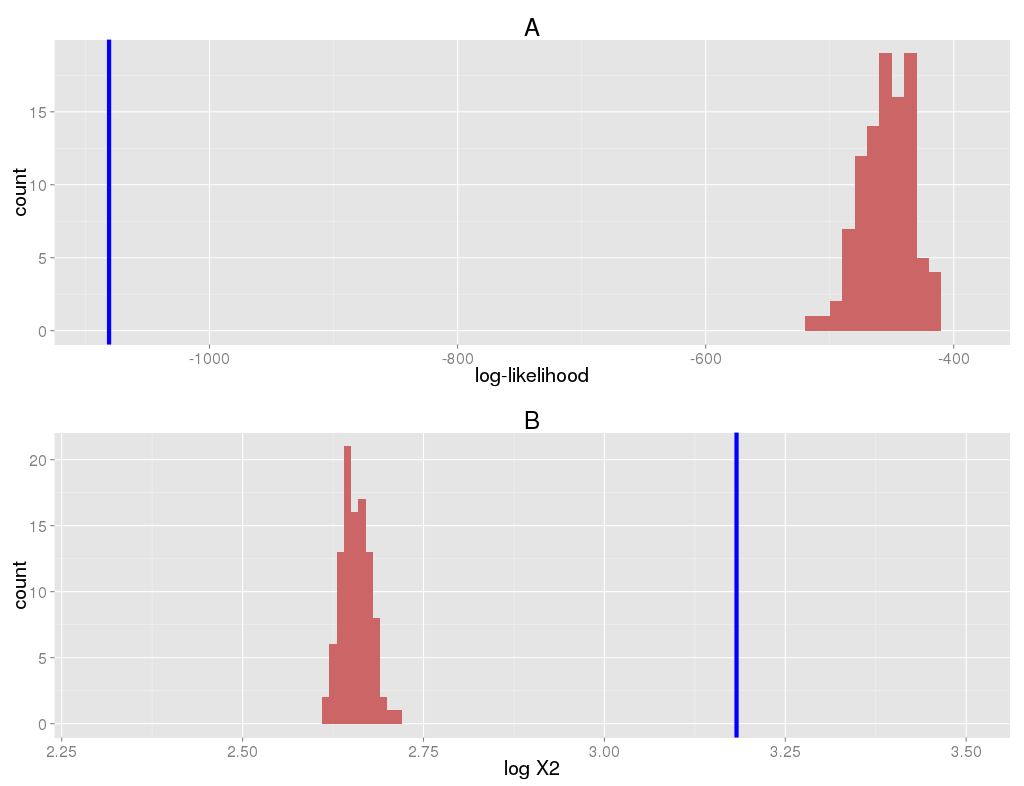


### Supporting Figure 4. Goodness-of-fit tests for DemNcd dataset based on 100 parametric bootstraps with better fits placed closer to zero. (A) Distribution of log-likelihoods for simulations (red) with value from real data set (blue). (B) Distribution of Pearson’s X2 statistic for simulations (red) with value from real data set (blue).


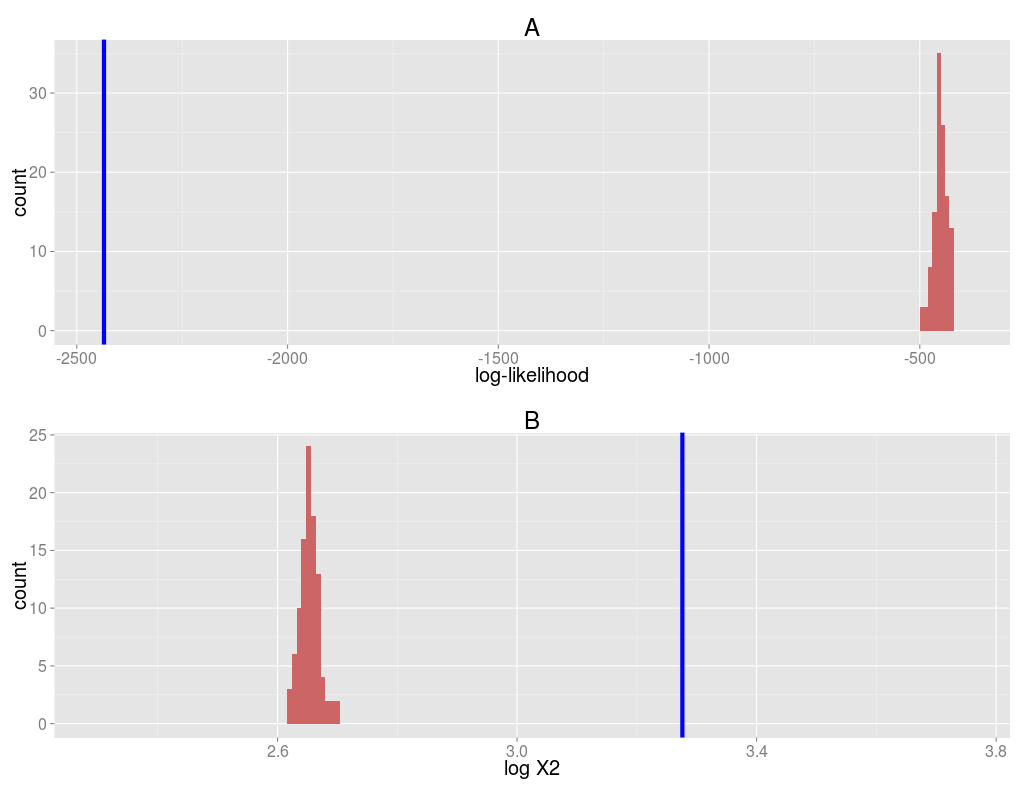


### Supporting Figure 5. SCGHET model conventional bootstrap results. Distribution of model’s parameters estimates based on 120 simulated DemSyn datasets, generated using maximum likelihood values from the real data (blue lines).


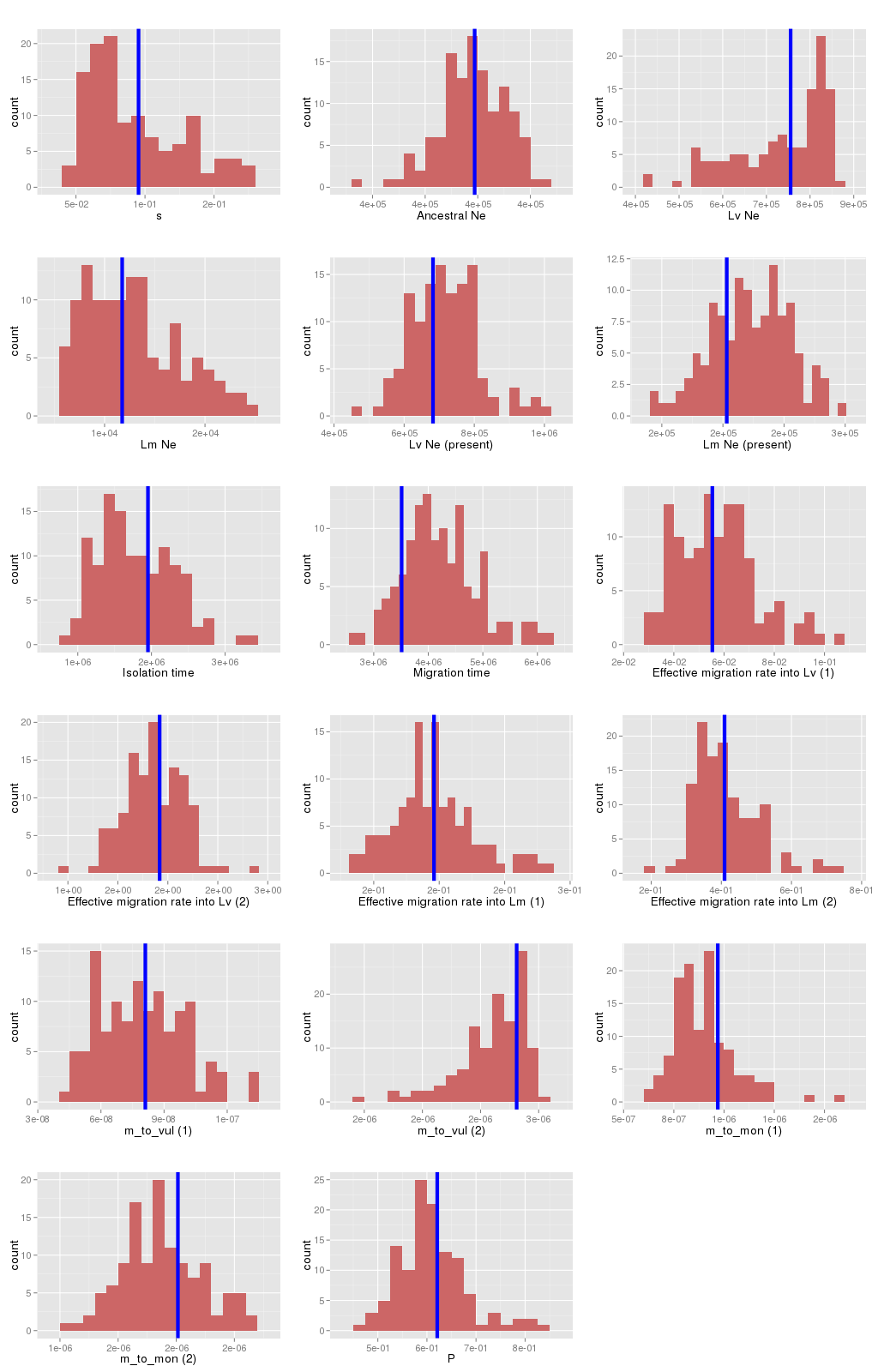


### Supporting Figure 6. SCGHET model conventional bootstrap results. Distribution of model’s parameters estimates based on 120 simulated DemNcd datasets, generated using maximum likelihood values from the real data (blue lines).

**
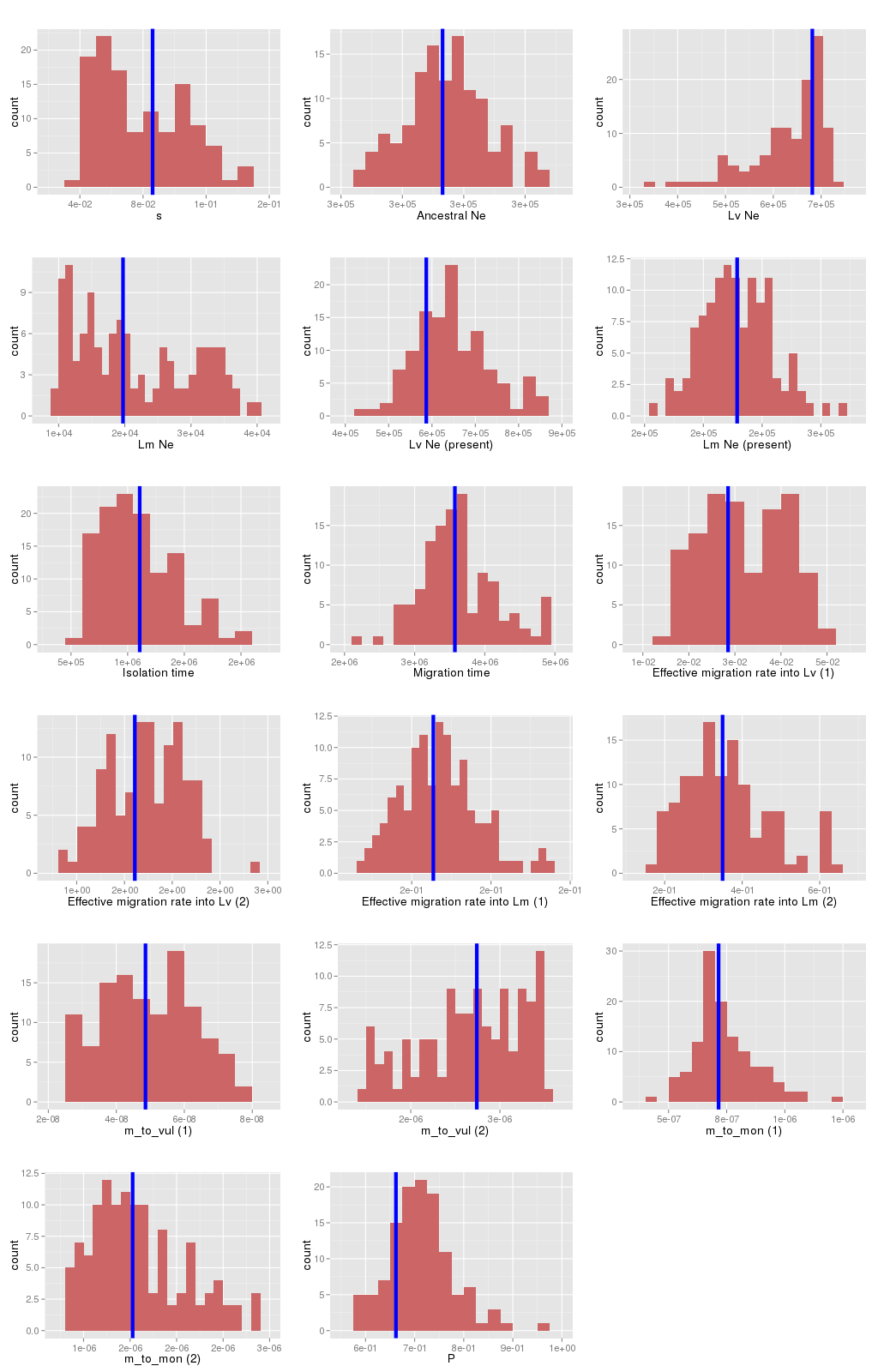
**
